# Wearable Liquid Metal Composite with Skin-Adhesive Chitosan–Alginate–Chitosan Hydrogel for Stable Electromyogram Signal Monitoring

**DOI:** 10.3390/polym15183692

**Published:** 2023-09-07

**Authors:** Jaehyon Kim, Yewon Kim, Jaebeom Lee, Mikyung Shin, Donghee Son

**Affiliations:** 1 Department of Electrical and Computer Engineering, Sungkyunkwan University, Suwon 16419, Republic of Korea; 2 Department of Intelligent Precision Healthcare Convergence, Sungkyunkwan University, Suwon 16419, Republic of Koreamikyungshin@g.skku.edu (M.S.); 3 Department of Biomedical Engineering, Sungkyunkwan University, Suwon 16419, Republic of Korea; 4 Department of Superintelligence Engineering, Sungkyunkwan University, Suwon 16419, Republic of Korea

**Keywords:** hydrogel, skin-adhesion, soft electronics, electromyogram, gesture classification, human–machine interface

## Abstract

In wearable bioelectronics, various studies have focused on enhancing prosthetic control accuracy by improving the quality of physiological signals. The fabrication of conductive composites through the addition of metal fillers is one way to achieve stretchability, conductivity, and biocompatibility. However, it is difficult to measure stable biological signals using these soft electronics during physical activities because of the slipping issues of the devices, which results in the inaccurate placement of the device at the target part of the body. To address these limitations, it is necessary to reduce the stiffness of the conductive materials and enhance the adhesion between the device and the skin. In this study, we measured the electromyography (EMG) signals by applying a three-layered hydrogel structure composed of chitosan–alginate–chitosan (CAC) to a stretchable electrode fabricated using a composite of styrene–ethylene–butylene–styrene and eutectic gallium-indium. We observed stable adhesion of the CAC hydrogel to the skin, which aided in keeping the electrode attached to the skin during the subject movement. Finally, we fabricated a multichannel array of CAC-coated composite electrodes (CACCE) to demonstrate the accurate classification of the EMG signals based on hand movements and channel placement, which was followed by the movement of the robot arm.

## 1. Introduction

Recently, there has been increasing interest in soft electronics in various fields, such as electronic skin [1,2,3], high-performance transistors [4,5], wearable skin patch biosensors [6,7,8], implantable healthcare devices [9,10,11], and human–machine interface applications [12]. In particular, research focused on the accurate control of prosthetics using wearable bioelectronics [13,14] was conducted frequently, which required stable and precise physiological signals from the body, especially electrocardiography (ECG), electromyography (EMG), and electroencephalography signals [15,16,17]. 

Previously, due to the noise caused by the stiffness mismatch between the conventional rigid electrode and the skin, it was challenging to use it in wearable devices. Therefore, various studies aimed to achieve stretchability by fabricating metal as a thin film. Nevertheless, limitations in terms of stiffness and low elasticity persisted [18,19]. As a solution, creating conductive composite electrodes by blending flexible materials with metals has become an important method for fabricating soft wearable healthcare devices. This approach allowed the electrode to maintain conductivity even in environments where the electrodes are under stretching conditions, enabling a more stable acquisition of physiological signals, similar to how human skin behaves. 

The use of a fully polymer-based composite is one method of enhancing the stretchability, biocompatibility, conductivity, and skin-matched modulus of electrodes [20,21]. These composites are usually prepared by mixing robust elastomers and polymers such as styrene–ethylene–butylene–styrene (SEBS), self-healing polymers (SHP), polyurethane (PU), and polydimethylsiloxane (PDMS) [22,23,24] with conductive fillers such as carbon nanotubes, graphene, Au, Ag, and liquid metal (LM) [25,26,27,28]. In particular, LM is frequently used because of its high thermal and electrical conductivity and excellent mechanical properties. LMs exist as single elements, such as mercury (Hg) and gallium (Ga), or as alloys like eutectic gallium-indium (EGaIn), which is a mixture of metal elements that behave like a fluid at room temperature and have a low melting point, low vapor pressure, and high surface tension. In addition, because they have fluidic properties similar to those of a liquid, composites fabricated using LM have good electrical conductivity during the stretching of the electrode compared to other solid-state metal fillers [29,30,31]. Moreover, the Ga-based LMs are biocompatible [32,33] which makes the LM composite a suitable electrode material for use as flexible wearable biosensors. However, the challenge of achieving a close interface between the device and skin for obtaining steady bio-signal measurements is yet to be addressed because of the detachment of the wearable device due to the movement of the subject while measuring biological signals from the body. Furthermore, when used in multichannel devices, the signals from each channel should be distinguished, which is critical for observing signals from different target parts of the body. To address this issue, a method is required to improve not only the interface of the device and skin but also the accuracy of the device placement at the target part to the target part to obtain stable signals.

Adhesive hydrogels are biomaterials that can be used to advance soft bioelectronics and overcome these problems. They provide good adhesion because of their gel-forming abilities, structures, and electrical charge [34,35,36,37]. Furthermore, their stretchability, flexibility [38], and self-healing ability [39,40] enhance their mechanical properties. Also, the biocompatibility and ionic conductivity of hydrogels [41,42] aid in collecting physiological signals such as EMG and ECG signals, which is the main advantage of the use of hydrogel-composite sensors and electrodes [43,44,45,46]. Chitosan and alginate are commonly used as adhesive hydrogels [47,48,49]. Chitosan is the product of the deacetylation of chitin, in which *N*-acetyl groups are removed. It consists of a long chain structure with β-(1→4)-linked *N*-acetyl-d-glucosamine units joined by glycosidic bonds. It has a high viscosity and does not dissolve in water or alcohol. It carries a strong positive charge in environments with pH < 6.5, allowing it to form ionotropic bonds with molecules bearing a negative charge. Because of these characteristics, it can be dissolved in HCl to create a hydrogel for various applications. Alginate, on the other hand, has a hexagonal six-carbon sugar structure. Found in the cell walls of seaweeds, it is hydrophilic and can form gels by binding to calcium ions. It remains stable at low temperatures and carries a negative charge due to its carboxyl groups. It can be dissolved in water to create alginate hydrogel. Chitosan and alginate are often used individually to form a single layer of hydrogel [50] or together to form a bilayer of hydrogel to enhance their adhesion to tissues or skin [51] with the electrostatic force between them.

After using these devices and obtaining more accurate bio-signals, further studies have been conducted in the fields of prosthetics and robotics [52]. The majority of these studies focused on the human–machine interface (HMI) for the control of robotic arms or prosthetics by recognizing hand gestures based on the changing inputs obtained from skin-attached strain sensors or EMG sensors [53,54,55]. The accuracy of controlling these devices can be improved by machine learning or the software design of microcontrollers. In addition, previous reports have suggested that the number of channels should be increased to collect sufficient information [56,57] or that appropriate muscles should be identified to distinguish each human hand gesture, which is an essential part of the precise classification of movements [58,59]. 

**Figure 1:**
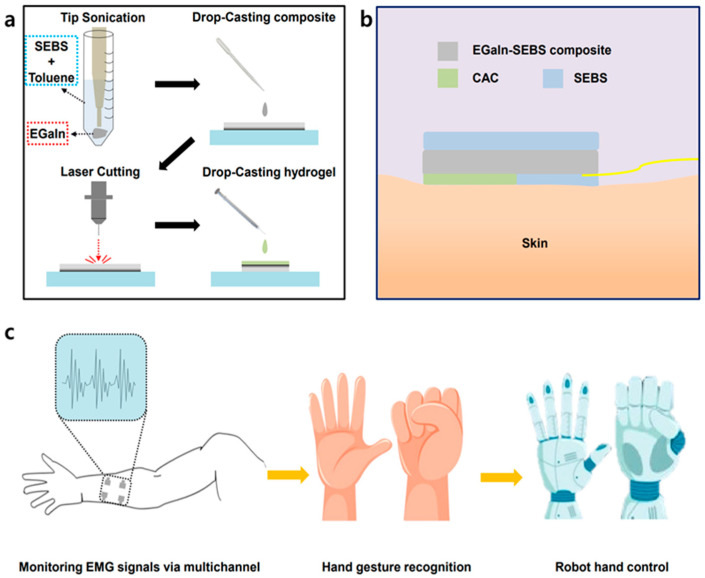
(**a**) Schematic illustration of the process of fabricating the CACCE. (**b**) Schematic of the EMG sensor on skin. (**c**) Schematic of the overall CAC hydrogel-coated multichannel EMG electrode measurement system.

In this study, we aimed to enhance the effective measurement of EMG signals and capture muscle activity using a three-layered hydrogel structure comprising chitosan–alginate–chitosan (CAC) as an adhesive interface between a soft composite electrode and the skin. The electrodes were fabricated using a composite of EGaIn, a commonly used LM, and SEBS, a stretchable elastomer. EGaIn was used in the form of sonicated particles as the liquid metal filler. The process of fabricating the EGaIn-SEBS electrode, which was coated with CAC hydrogel, is presented in Figure 1a, in the order of tip sonication, drop-casting of the EGaIn-SEBS composite, laser cutting, and drop-casting of the CAC hydrogel. The EMG-sensing electrode device placed on the skin is presented in Figure 1b. The CAC-coated composite electrode (CACCE) exhibited sufficient adhesion to the skin, thereby fixing the electrode during various dynamic actions. As shown in the figure, the CAC hydrogel behaved as an adhesive interface between the EGaIn-SEBS electrode and the skin, which was encapsulated by the SEBS film. Encapsulation can be conducted in various materials, such as PDMS and polyimide, and methods like spin coating, spray coating, and direct diffusions [60]. Because SEBS can be mechanically combined with another SEBS film, we chose SEBS as an encapsulation material for the efficient conjunction and homogeneous structure of the device. Furthermore, we developed a flexible, stretchable, multichannel EGaIn-SEBS composite electrode array of the CACCE to classify the EMG signals according to different hand movements, thereby facilitating the implementation of an HMI that mimics human hand gestures via the control of a robotic arm. A schematic of the system is presented in Figure 1c. The substrate and electrodes of the device comprised both flexible and stretchable materials, thereby providing the advantages of comfortable wearability and effective signal measurement and enabling a more accurate classification of hand movements with the help of the CAC hydrogel.

## 2. Materials and Methods

### 2.1. Preparation of EGaIn-SEBS Composite

The EGaIn-SEBS composite film was prepared using the following procedure. First, 180 mg of SEBS (Tuftec™ H1062, Asahi Kasei Co., Chiyoda, Tokyo, Japan) was dissolved in 2 mL of toluene (Sigma-Aldrich, St. Louis, MO, USA) by stirring for 6 h at 200 rpm. Subsequently, 700 mg of EGaIn (12478, Alfa Aesar, Haverhill, MA, USA) was added to 2 mL of SEBS solution in a conical tube (Dongnam Chemical, Seoul, Republic of Korea). To homogenize the particles, the composite solution was subjected to tip sonication using a VCX–750 (Sonics & Materials Inc., Newtown, CT, USA) operating at a power of 300 W power for 10 min. The resulting EGaIn-SEBS solution was then drop-cast onto 3 cm × 3 cm octadecyl trichlorosilane (OTS)-treated glass substrates and left to evaporate at room temperature for 6 h. Finally, the composite was peeled off from the glass surface to complete the fabrication process. The LM particles in the drop-cast composite solution sank to the bottom during the evaporation process, thereby making the under and upper sides of the composite conductive and insulating parts, respectively. 

### 2.2. Mechanical and Electrical Characteristics of EGaIn-SEBS Composite

We analyzed the microscopic structure of the composites using field emission scanning electron microscopy (FE-SEM; JSM-IT800, JEOL, Peabody, MA, USA). The size of the composite sample was 3 mm × 3 mm to observe its structure from the top view. Another sample was prepared by cutting the composite using a razor blade with a height of 3 mm and a width of 1 mm, which was stretched to 100% of its original length. All the samples were attached to a chuck using double-sided carbon tape (T01-117-355, Lklab Korea, Namyangju-si, Gyeonggi-do, Republic of Korea).

A potentiostat (ZIVE SP1, WonATech Co., Seoul, Republic of Korea) was used to measure the electrochemical impedances of the composites. The dimensions of the composite samples were 1 cm × 1 cm. The electrode was placed on a 2 cm × 2 cm SEBS substrate, which was prepared by dissolving 180 mg of SEBS in 1 mL of toluene, followed by drop-casting on a 2 cm × 2 cm glass substrate and evaporation at room temperature. A stripped wire with an outer diameter of 0.45 mm was attached to the composite electrode via the encapsulation of Tegaderm film (3M, Maplewood, MN, USA). The other end of the stripped wire was soldered to a 1 cm × 1 cm gauge filler, which was connected to the alligator clip of the wire of the potentiostat. The samples of the EGaIn-SEBS composite electrode, Ag/AgCl reference electrode, and platinum counter electrode were immersed in phosphate-buffered saline (1X PBS Buffer, pH 7.2, Biosesang, Yongin-si, Gyeonggi-do, Republic of Korea). Another CACCE sample was used to compare the electrochemical impedance of the bare composite electrode with that of a hydrogel-coated electrode. The CACCE was coated with a 200-μL layer of chitosan and alginate each. The frequency range of the impedance was 1 Hz to 100 kHz, and the amplitude was 10 mV.

Meanwhile, a universal testing machine (UTM, Instron 34SC-1, Seoul, Republic of Korea) was used to evaluate the mechanical properties of the samples. The EGaIn-SEBS composite samples were cut to a width of 5 mm and length of 3 cm. The samples were fixed to the chuck by sealing them between an A4 paper of size 2 cm × 2 cm, and they were also stuck together using double-sided tape (3M, Maplewood, MN, USA). They were loaded onto a chuck with an initial length of 3 mm, which was stretched at a speed of 20 mm∙min^−1^. Bare SEBS films of the same size as the composite samples were loaded onto a chuck using the aforementioned method. Bare SEBS films were fabricated using the SEBS–Toluene ratio of 180 mg: 2 mL, with a thickness of 80 μm, whereas the composite sample was 140 μm thick. All the measurement data were obtained using Instron Bluehill software.

Furthermore, a 3 mm × 3 mm size of EGaIn-SEBS composite was prepared for cyclic testing. A digital multimeter (Keithley 2450 SourceMeter, Tektronix, Inc., Beaverton, OR, USA) and a motor-based x-axis stretcher (SMC-100, Jaeil Optical Corp., Seoul, Republic of Korea) were used for the test. A total of 600 cycles were conducted, where 50% strain was applied at a rate of 1 mm per second, followed by a return to the original state. 

### 2.3. Preparation of CAC Hydrogel

Chitosan and sodium–alginate powders (medium viscosity) were purchased from Sigma-Aldrich to produce the chitosan–alginate–chitosan three-layered hydrogel. Twenty milligrams of chitosan and alginate powder each were separately dissolved in 1 mL of 0.05 M HCl and deionized water (10 wt%), respectively, which was followed by stirring for 6 h at 200 rpm to obtain the hydrogel. Next, 450 μL of the dissolved chitosan solution was drop-casted onto the 1.5 cm × 1.5 cm plastic mold and evaporated at room temperature for 18 h to obtain the first chitosan layer. Subsequently, the drop-casting and evaporation steps were repeated using alginate, which was followed by chitosan to create the second and third layers of the hydrogel film, respectively. By sequentially depositing chitosan and alginate layers, a chitosan–alginate–chitosan three-layered hydrogel was successfully fabricated. The finished CAC film was carefully peeled off from the plastic mold. The peeled CAC film was cut using a razor blade to the size of 1 cm × 1 cm, which was the same as that of the electrode. The CACCE could be fabricated either by drop-casting the chitosan and alginate solution directly or by placing the dried CAC hydrogel film on the composite electrode and dropping the PBS onto it to coat the hydrogel on the electrode appropriately. 

### 2.4. Adhesion Characteristics of CAC Hydrogel

To evaluate the adhesion of the hydrogel, a three-layered CAC hydrogel film was prepared by drop-casting 1.25 mL of each solution. After the fabrication of the CAC–layer hydrogel film, it was peeled off the mold, thereby completing the preparation of the CAC film. Two pieces of a T-shaped plastic substrate were fabricated using 3D printing. Subsequently, a 1 cm × 1 cm overhead projector (OHP) film (CopierLand, Paju-si, Gyeonggi-do, Republic of Korea) was fixed to the T-shaped plastic substrate using double-sided tape. Finally, the rat skin was fixed to the OHP film by spreading super glue with a cotton swab, which was coated with the CAC hydrogel by placing the hydrogel film on the rat skin and dropping one drop of PBS onto it. The coated rat skin on each T-shaped substrate was adhered to another rat skin. The UTM was used to pull two glued rat skins at a velocity of 20 mm/min. The maximum adhesion strength of the CAC three-layered hydrogel film was compared to that of the chitosan single-layered hydrogel film and chitosan–alginate bilayer, wherein 1.25 mL of the chitosan and alginate solution was dropped into the plastic mold, thereby demonstrating superior adhesion. The measurement data were acquired using Instron Bluehill software.

### 2.5. Fabrication of Customized Electrode Array

To fabricate a customized electrode pair, the initial step involved the preparation of an EGaIn-SEBS composite film. The composite electrode was then patterned using an optical fiber laser marker (Hyosung Laser, Bucheon-Si, Gyeonggi-do, Republic of Korea) over 25 cutting cycles. The pattern of the electrode, which was designed using AutoCAD, was in the shape of a bottle of size 1 cm × 1 cm and a mouth of 5 mm × 5 mm. Subsequently, the patterned electrode was coated with three layers of the CAC hydrogel to form the CACCE. For the SEBS substrate, a solution was prepared by dissolving 180 mg of SEBS in 1 mL of toluene. This solution was drop-cast onto a 3.5 cm × 3.5 cm glass substrate and allowed to evaporate for 6 h, thereby resulting in the formation of the SEBS substrate. Next, after fixing the patterned electrode on the SEBS substrate, the electrode was connected to a stripped wire with the encapsulation of the thin SEBS film. A thin SEBS film was prepared by dissolving 180 mg of SEBS in 2 mL of toluene. The stripped wire was attached to the patterned electrode while ensuring that the thin SEBS film encapsulated the connection.

### 2.6. Measurement Processing of EMG Signals Obtained from Human Skin

For the efficient measurement and monitoring of EMG signals, a Tegaderm film was used for EMG recording with the EGaIn-SEBS electrodes fixed to the skin. The conductive side of the composite electrode was exposed to the skin. The signals were recorded using a biosignal amplifier (Bio Amp FE231, AD Instruments, Dunedin, New Zealand) and a data acquisition device (PowerLab 8/35, AD Instruments Springs, Colorado Springs, CO, USA). We also used LabChart 8 Pro (AD Instruments, Bella Vista, NSW, Australia) to obtain the EMG data. Moreover, action potential signals were filtered based on the International Society of Electrophysiology and Kinesiology standard (25 to 50 Hz band-pass filter). Ten drops of PBS were subjected to CACCE to achieve better hydrogel adhesion via swelling, which helped the electrode stick to the skin. The ground electrode was attached to the elbow, and the reference electrode (RE) and sensing electrode (SE) were attached to the forearm by hanging a hook-type electrode using a stripped wire. To demonstrate the entire device with multiple channels, two pairs of RE and SE were placed on the flexor pollicis longus, and the other two pairs of RE and SE were placed on the flexor digitorum superficialis to obtain different signals for each muscle [61]. The three distinguished actions of clenched fist (CF), finger extension (FE), and finger relaxation (FR) were performed for 10 s each to analyze the EMG signals according to each gesture. The mean of the acquired EMG data for 10 s entered the input of a conventional microcontroller (Arduino UNO R3) that moved the robot’s hand (DFRobut bionic robot hand ROB0142, Shanghai, China). The authors obtained approval from the Institutional Review Board (approval no. SKKU 2023-07-045) of Sungkyunkwan University for measuring the EMG signals.

## 3. Results and Discussions

### 3.1. Characteristics of CAC Hydrogel

**Figure 2:**
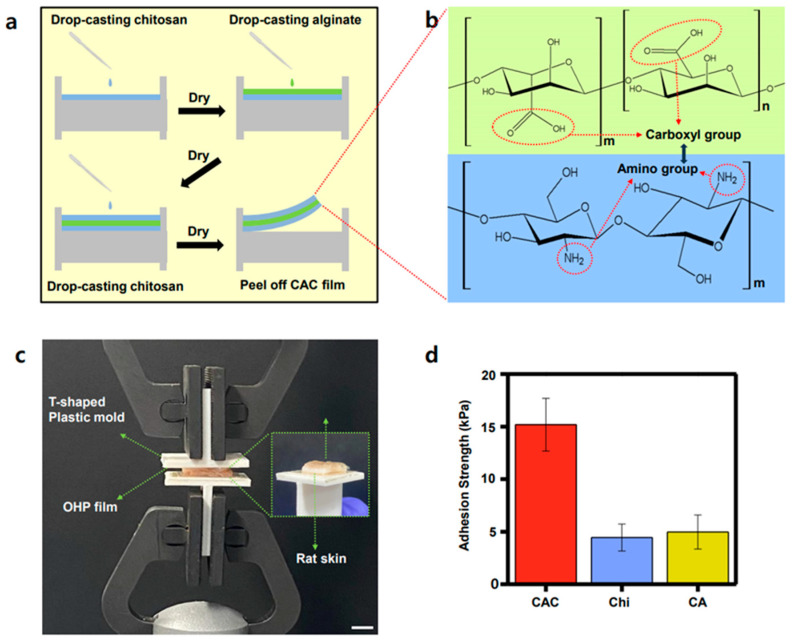
(**a**) Illustration of the fabrication process of suggested CAC three-layered hydrogel film. (**b**) Chemical structures of chitosan (bottom) and alginate (top). (**c**) Photograph of the measurement process of CAC hydrogel adhesion strength. (scale bar: 1 cm) (**d**) Bar graph of the adhesion strength of CAC three-layered hydrogel film, chitosan single-layered hydrogel film, and CA bilayer hydrogel film observed on the coating each of them on rat skin.

The monitoring and measuring of physiological signals are essential for assessing human health status and providing appropriate feedback. In the case of wearable healthcare devices, extensive research was conducted to enhance the comfort and conformal contact between soft devices and the skin by developing electrodes with suitable physical properties that match those of human skin. However, because the electrode lacks adhesive properties, this results in device slippage or significant noise in the signal measurement. To address this issue, methods involving the use of hydrogels as adhesive materials between the device and skin are proposed to improve the adhesion between the electrode and skin, thereby enhancing the reliability and accuracy of the measured signals. Therefore, enhancing the adhesion between the skin and the electrode by coating the EGaIn-SEBS electrode with a three-layered hydrogel of CAC is suggested. The fabrication process for the individual CAC films is presented in Figure 2a. Chitosan is a biopolymer derived from shrimp and crab, containing glucosamine units in its polysaccharide structure, which exhibits cationic properties because of the presence of amino groups in its polymer chain (Figure 2b, bottom). However, alginate, a natural polysaccharide found in seaweed, exhibits anionic properties because of the presence of carboxylic groups in its polymer chain. It also possesses excellent gel-forming abilities and is often used in combination with chitosan to create composite materials (Figure 2b, top). Consequently, the combination of chitosan and alginate in the CAC hydrogel forms a stronger bond through electrostatic forces compared to a single-layered hydrogel. Furthermore, the skin tissue, which primarily carries a negative charge, exhibits better adhesion to chitosan, which has a positive charge. Therefore, we used a three-layered CAC structure to realize a higher adhesion strength and toughness. This structure has high absorptivity and adhesion due to the conjunction between the two hydrogel interfaces [51]. Moreover, to assess the adhesive strength of the CAC three-layered hydrogel structure, we coated the CAC film between the rat skin and measured the adhesive strength using a UTM, which resulted in significantly higher values of strength (15.18 kPa) compared to those obtained using a single layer of chitosan hydrogel film (4.44 kPa) or chitosan–alginate (CA) bilayer film (4.97 kPa) (Figure 2c,d).

### 3.2. Characteristics of EGaIn-SEBS Electrode and CACCE

**Figure 3:**
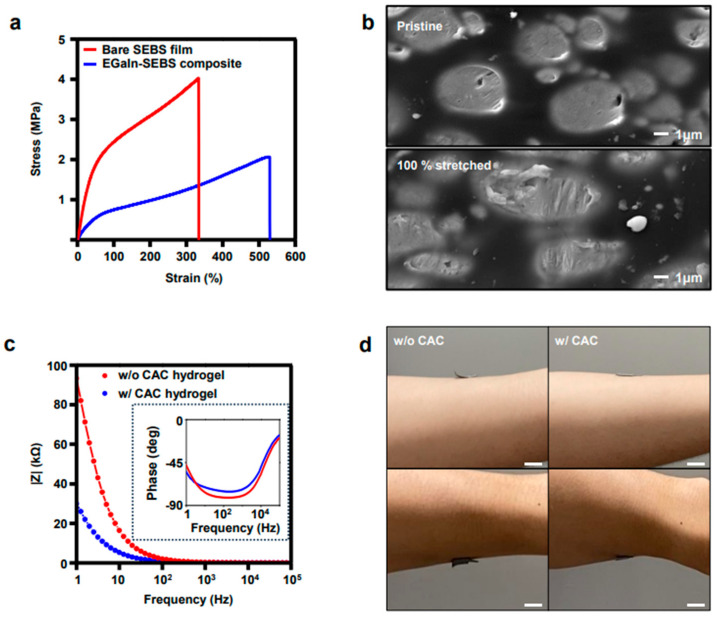
(**a**) Stress–strain curves of EGaIn-SEBS composite and bare SEBS. (**b**) SEM image of pristine EGaIn-SEBS composite electrode (top) and 100% stretched electrode (bottom). (scale bar: 1 μm) (**c**) Impedance measurement of composite electrodes with hydrogel (CACCE) and without hydrogel. (**d**) Comparison of the adhesion of CACCE and bare composite EGaIn-SEBS composite electrode by observing the conformal contact of them to the skin during the movement of the subject arm. (scale bar: 1 cm).

The EGaIn-SEBS electrode was fabricated using the tip sonication method by converting EGaIn in the dissolved SEBS solution into particles, followed by drop-casting, evaporation at room temperature, and laser cutting. Previous studies showed that various elastomers and liquid–metal composite electrodes [62] exhibited excellent stretchability and conductivity. The endurance of the electrode is illustrated by the stress–strain curves in comparison with those of the bare SEBS elastomer, as shown in Figure 3a. The liquid–metal composite exhibited superior stretching properties by withstanding a maximum value of 520% stretching compared to bare SEBS, which endured 340% stretching. This result suggests that the EGaIn-SEBS composite was softer than the bare SEBS film, which was sufficient for wearable electrodes. Furthermore, the cyclic test results in Figure S1 reveal minimal variations in the resistance throughout the cycles. Given that the maximum strain of human skin was approximately 25%, considering the intent to employ the composite electrode in wearable devices, the experimental data suggests that the composite electrode can maintain signal stability even in environments where strain is repeatedly applied hundreds of times.

In addition, the SEM image showed that EGaIn was scattered homogeneously in the SEBS composite (Figure 3b), which indicates that the composite was conductive over its entire surface. Similarly, the microscopic structure of the EGaIn-SEBS composite stretched to 100% exhibited not only evenly distributed particles but also their stretched shape, which explains the conductivity and mechanical durability of the electrode despite its stretching. In addition, the electrical and adhesive characteristics of the CACCE were observed. Figure 3c presents the electrochemical impedance measurements of the CACCE and bare EGaIn-SEBS electrode without the hydrogel. The electrodes with hydrogel exhibited greater stability and lower impedance compared to those without hydrogel. At high frequencies, the impedance of the CACCE was very low because the capacitance of the hydrogel increased. Thus, the ions and polymer chains could move freely. As the frequency decreased, the capacitance decreased, thereby increasing the impedance. In particular, in the frequency range of 1 to 100 Hz, which is a typical frequency range for EMG signals when relatively static movements of physical activities are made, CACCE exhibited a lower impedance than the composite electrodes without the hydrogel due to the ionic conductivity of the CAC hydrogel. In addition, the phase degree of the impedance shows that the capacitance of the device had a smaller effect on the impedance from 1 to 100 Hz. This result indicates that the hydrogen-coated electrodes collected more stable and accurate physical signals than those in the absence of the hydrogel. The electrode pair was adhered to the arm to guarantee stable measurements and wearability. The hydrogel helped the electrode pair stick to the arm during several dynamic physical movements (Figure 3d), whereas the absence of the hydrogel resulted in inadequate adhesion and unconformable contact under identical conditions, which led to the detachment of the electrode.

### 3.3. Demonstration of HMI with Multichannel EMG Electrode Array for Classification of Motions

**Figure 4:**
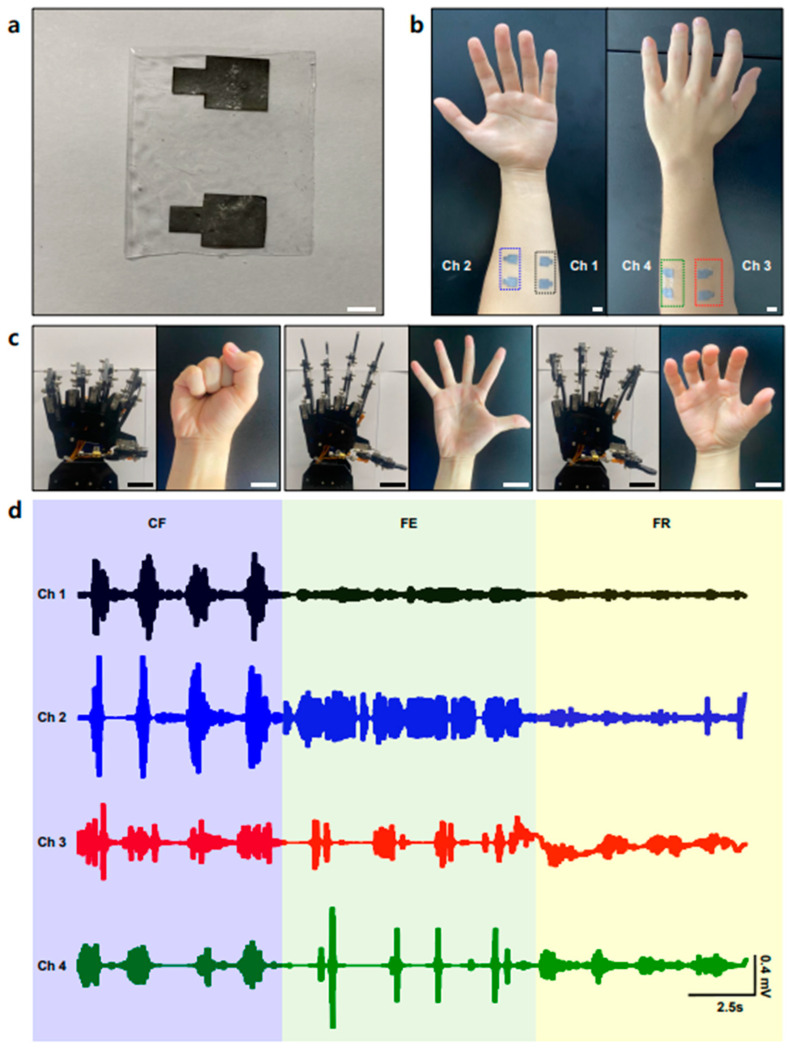
(**a**) Optical image of fabricated multichannel electrode pair. (scale bar: 1 cm) (**b**) Optical image of the multichannel electrode on a human arm. (scale bar: 1 cm) (**c**) Human actions (right) of the clenching fist (Action 1), finger extension (Action 2), finger relaxation (Action 3), and corresponding robot arm movements (left). (scale bar: 5 cm) (**d**) EMG signals of each channel for various human motions.

Flexible electrodes are used in HMI as an application of soft electronics because they acquire more precise signals from the human body. Recent studies have focused on the design of flexible and stretchable electrodes using novel conductive materials and advanced fabrication techniques. Signal processing algorithms have also made advances via not only machine learning but also deep learning methods such as DNN and CNN, which can be demonstrated as neuromorphic systems [63,64,65], thereby enabling the accurate interpretation of signals for the delicate control of robotic systems via intuitive gestures. Using a multichannel electrode to obtain various EMG signals and classify different gestures is another method for improving the HMI [66,67]. Based on our previous confirmation of the conductivity and adhesive properties using CACCE, we developed a multichannel electrode array for signal processing. This facilitated signal processing and analysis on multiple channels, thereby enhancing the capability of our system to capture and interpret complex muscle activity patterns and distinguish gestures of the human hand. To demonstrate the capabilities of the multichannel EMG electrode array, we designed and prepared four pairs of sensing electrodes and reference electrodes measuring 1 cm × 1 cm. An image of the composite electrode pair is shown in Figure 4a. These electrodes were attached to different target muscles of the arm (Figure 4b). Channels 1 and 2 were attached to the flexor pollicis longus (front part of the forearm), whereas Channels 3 and 4 were attached to the flexor digitorum superficialis (back part of the forearm). Subsequently, we conducted EMG signal measurements for three distinct actions(Figure 4c): fist clenching (Action 1, left), finger extension (Action 2, middle), and finger relaxation (Action 3, right), which are commonly used motions in daily life. Each of the motions was demonstrated four times during each section of 0–10 s, 10–20 s, and 20–30 s. The corresponding EMG signals obtained from the channels (Channels 1–4) are presented in Figure 4d, which illustrates the variations in the EMG signals across different actions. The mean values of the acquired EMG data of each channel were sent to the Arduino UNO, and the threshold of each gesture was set, thereby facilitating the development of a robotic arm capable of mimicking human movement. When the microprocessor recognized the EMG signal in the three cases of CF, FE, and FR, it adjusted the angle of the servomotor of the corresponding thumb to the little fingers of the robotic arm. Such a demonstration of the use of robotic arms with multichannel electrode devices encourages the further use of soft bioelectronics in prosthetics with the use of more physiological information and improved machine-learning methods, resulting in the accurate classification of movements of the human body. 

## 4. Conclusions

In this study, we fabricated flexible and stretchable EGaIn-SEBS electrodes and a CAC three-layered hydrogel to achieve conformal contact with human skin and realize the efficient measurement of EMG signals. The EGaIn-SEBS electrode exhibited excellent stretchability by enduring 520% strain and conductive properties because of the evenly distributed liquid–metal filler, which could be observed in the SEM image and stress–strain curves obtained using the UTM. The CAC hydrogel exhibited a maximum adhesion strength of 15.18 kPa compared to a single layer of chitosan hydrogel of 4.44 kPa and CA bilayer hydrogel film of 4.97 kPa when coated onto the rat skin. Moreover, the CACCE provided superior impedance data at low frequencies of 1 to 100 Hz due to the CAC’s ionic conductivity and greater adhesion stability of the CAC hydrogel compared to the electrodes without the hydrogel. This was confirmed by observing the attachment of the electrode to the skin during the movement of the subject. This improvement can be attributed to the enhanced attractive force inside the hydrogel due to the oppositely charged amino and carboxyl groups, which are the chemical components of chitosan and alginate, respectively. Furthermore, we successfully demonstrated the effectiveness of a multichannel electrode array in the precise processing of EMG signals and in accurately classifying various hand gestures associated with various target muscles. When the fist was clenched (CF), Ch2 was especially activated by a maximum amplitude of 0.7 mV, which was 0.6 mV at Ch4 when the fingers were extended (FE) and 0.2 mV at Ch3 and 4 when the fingers were relaxed (FR). The resulting data were then used to control the movement of the robotic arm, thereby demonstrating the potential of our approach for HMI applications. Therefore, we believe that the multichannel of CACCE is an efficient way to collect various EMG data and classify the motions of the human body. Furthermore, integrating these flexible sensors that can collect more stable and better-quality signals with various machine-learning algorithms could help us take one more step to enhance HMI and prosthetic systems.

## Data Availability

Not applicable.

## Supplementary Materials

The following supporting information can be downloaded at: https://www.mdpi.com/article/10.3390/polym15183692/s1, Figure S1: Cyclic stretching test of the EGaIn-SEBS composite under 50% strain and enlarged part of the graph (inset).
