# Supplementary material for: Wearable Liquid Metal Composite with Skin-Adhesive Chitosan–Alginate–Chitosan Hydrogel for Stable Electromyogram Signal Monitoring
Source: Polymers (Basel). 2023 Sep 7;15(18):3692. doi: 10.3390/polym15183692 (PMC10536051; doi:10.3390/polym15183692)
Supplement: Supplementary file 1 [file polymers-15-03692-s001.zip › polymers-2584005-supplementary.pdf]

Supplementary Information

Wearable liquid metal composite with skin-adhesive chitosan-alginate-chitosan hydrogel for stable electromyogramsignal monitoring

Jaehyon Kim†, Yewon Kim, Jaebeom Lee, Mikyung Shin*, Donghee Son *

†These authors contributed equally to this work

*Correspondence to be addressed to: *daniel3600@g.skku.edu*

This PDF file includes:

Supplementary Figure. 1

Supplementary Figure

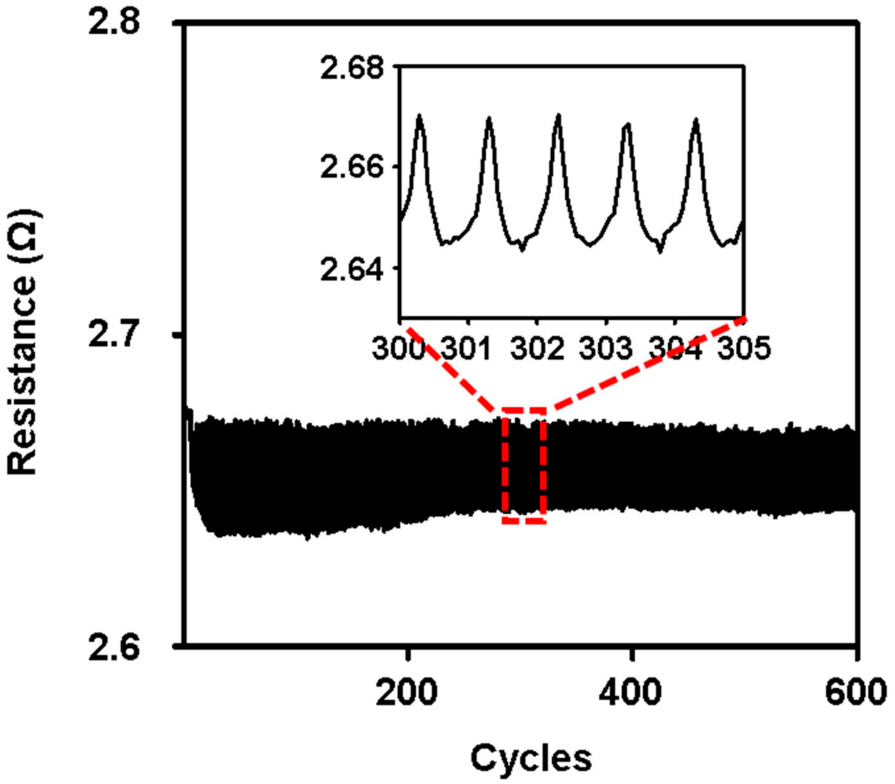

Figure S1. Cyclic stretching test of the EGaIn-SEBS composite under 50% strain and enlarged part of the graph (inset).
